# Quantity and Quality of Dairy Product Intake and Their Relationship with Body Composition in Children and Adolescents from Mexico City

**DOI:** 10.3390/nu17162705

**Published:** 2025-08-21

**Authors:** Armando Partida-Gaytan, Diana Montiel-Ojeda, Patricia Clark, Desiree Lopez-Gonzalez

**Affiliations:** 1 Clinical Research Direction, Hospital Infantil de México Federico Gómez, Mexico City 06720, Mexico; dr.partida.g@gmail.com; 2 Faculty of Medicine, Universidad Nacional Autonoma de Mexico, Mexico City 04510, Mexico; diana-montiel@facmed.unam.mx (D.M.-O.); clark@unam.mx (P.C.); 3 Clinical Epidemiology Research Unit, Hospital Infantil de México Federico Gómez, Mexico City 06720, Mexico

**Keywords:** body composition, milk-derived products, dairy products, children, adolescents

## Abstract

**Background**: The association between milk-derived or dairy product intake (DPI) and the body composition (BC) of children and adolescents continues to be controversial. **Objective**: This study sought to evaluate the association between the quantity and quality of DPI and the BC of Mexican children and adolescents. **Methods**: A secondary data analysis of a cross-sectional population-based sample of 2104 children and adolescents (aged 4–18 years) recruited from schools in Mexico City was performed. To assess the association between the quantity and quality of DPI, evaluated by questionnaire, and BC, dual X-ray densitometry (DXA) and surrogate parameters of the fat mass index (FMI) and lean mass index (LMI) were used. The adequacy of the quantity and quality of DPI was classified according to age-specific recommendations and the sugar, sodium and fat contents of the products consumed. Relationships were assessed by means of lineal regressions adjusted for age, sex, physical activity, sleep time and screen hours. **Results**: We included a total of 1840 participants aged 4.5–18 years, 52% of whom were female. Average daily DPI was 4.0 ± 2.4 servings/day, with a predominance of milk without sugar (42.7% of DPI). The quantity and quality of DPI significantly reduced the FMI (beta = −0.1 kg/m^2^, 95% CI −0.17 to −0.06, *p* < 0.001 and beta = −0.17 kg/m^2^, 95% CI −0.26 to −0.07, *p* < 0.001). No significant relationships were seen with the LMI. **Conclusions**: Higher quantities of good-quality DPI are associated with lower adiposity in Mexican children and adolescents.

## 1. Introduction

Dairy products are milk-derived products (including cheese and yogurt) which have ancestral origins that can be traced back to the start of human feeding practices [1]. During childhood and adolescence, dairy product intake (DPI) is important because such products represent balanced sources of energy, proteins and several micronutrients (calcium, magnesium, zinc, iodine, phosphorus and vitamins A, D, K and B12) [2,3,4]. According to the Dietary Guidelines for Americans 2020–2025 (DGA 2020–2025), dairy products include fat-free or low-fat milk, yogurt, cheese, lactose-free milk or yogurt and alternatives such as fortified soy beverages, although the latter are not milk-derived products. The recommended DPI varies from 2.5 cups for children aged 2–8 years to 3 cups for children and adolescents aged 9–18 years (1 cup = 240 mL or 8 oz) [5,6].

In Mexico, the official dietary guidelines, primarily established through the “Mexican Official Standard, Basic health services. Promotion and education for health in food. Criteria for giving orientation NOM-043-SSA2-2012 [7]”, promote proper nutrition and health education via food group recommendations presented as visual representations similar to MyPlate (USA) or the Eatwell Plate (UK). These guidelines are complemented by the “2015 Food and Physical Activity Guidelines”, which emphasize balanced eating, water intake and regular physical activity. However, none of these documents is specific about the number of servings recommended per food group.

The health benefits associated with balanced DPI have been widely reported and include increased bone mineral density, lower risk of fractures, decreased cardiovascular risk, weight control, reduced likelihood of being overweight and prevention of obesity, as well as better body mass distribution [8,9,10,11,12,13,14,15]. However, the quality of certain dairy products has been demonstrated to modify this effect, with research associating a higher intake of saturated fats, sugars or highly processed dairy products with various metabolic implications [13,16].

Research conducted within the Mexican population has indicated a decline in the quality of dairy products, with an increase in sugar content being observed in flavored beverages, including flavored milks and drinkable yogurts [17,18]. This phenomenon is of concern, as Mexico’s child population exhibits one of the highest global rates of obesity, with one in three children being overweight and one in four suffering from obesity. This underscores the imperative for comprehensive dietary assessment in children and adolescents, a crucial yet formidable challenge for the public health sector [19].

In this context, it is necessary to consider both the quantity and the quality of dairy products consumed by this population, to assess the potential impact on body composition and metabolism in children. Therefore, the aim of this study is to evaluate the associations between the quantity and quality of DPI and the body composition (BC) of Mexican children and adolescents.

## 2. Materials and Methods

### 2.1. Study Participants

Data for the current analysis came from the “Reference Values of Body Composition in Pediatric Mexican Population” study, a population-based cross-sectional study of children and adolescents from Mexico City [20]. Subjects were recruited from public and private schools in Mexico City through a randomized, stratified, multistage procedure to represent this population. Written invitation was sent to parents between March 2015 and November 2019. Family members and friends who met the inclusion criteria were also invited.

For such study, the inclusion criteria included participants aged between 4 and 20 years that did not have any known chronic, endocrine, systemic, respiratory, neurological, cardiac or psychiatric disorders or any chromosomal diseases, genopathies or dysmorphic syndromes and that provided informed consent/assent. For this study, we only included subjects aged between 4 and 18 years.

### 2.2. Study Procedure

All assessments, including questionnaires and measurements, were performed at the Clinical Epidemiology Research Unit at the Hospital Infantil de México Federico Gómez by the same team of certified pediatricians and nutritionists throughout the study. Participants attended an appointment at the unit after fasting for 8 h, and were accompanied by a parent and/or legal guardian. The participants were clinically and nutritionally assessed the same day.

This study was reviewed and approved by our Institutional Research, Ethics and Biosafety Committees (registry no. HIM 2015–055).

### 2.3. Measurements

Clinical measurements of subjects included weight and height measurements taken with the subjects wearing lightweight clothing using a SECA^®^ 284 (Seca GmbH & Co., Hamburg, Germany) scale with a stadimeter. The measurements were standardized and performed by trained nutritionists. The body mass index (BMI) was calculated as the weight (kg) divided by the square of height (m) [21]. The subjects were further classified according to their BMI percentile value, based on the growth charts of the World Health Organization (WHO), as underweight (<5th percentile), healthy weight (5th to <85th percentile), overweight (85th to <95th percentile) or obese (≥95th percentile) [22].

#### 2.3.1. Body Composition Measurements by Dual X-Ray Absorptiometry (DXA)

A whole-body scan was performed on all participants using a Lunar-iDXA densitometer (GE Healthcare^®^, Madison, WI, USA) according to the manufacturer’s instructions and analyzed using ENCORE^®^ software version 15. Calibration of the densitometer was performed on a weekly basis according to the manufacturer’s instructions, and measurements were performed by an International Society of Clinical Densitometry (ISCD)-certified nurse. DXA total body composition assessment with regional analysis provided data for total body (with head) fat mass (FM), lean soft tissue mass (LM) and bone mineral content (BMC) [23], and regional data from the arms, legs and trunk [24]. DXA FFM values were calculated as total body LM plus BMC. The fat mass index (FMI) was calculated as total body fat (kg) divided by the square of height (m), and the lean mass index (LMI) as total lean mass (LM) (kg) divided by the square of height (m).

#### 2.3.2. Diet Assessment

Data on quantity and types of food and beverage intake were collected by trained nutritionists through a structured interview with each subject using two 24 h recall surveys, one regarding the participant’s most recent typical school day and the other the participant’s most recent typical weekend day. A weighted mean from both surveys was calculated for the corresponding analyses. A validated 12-month food frequency questionnaire (FFQ) was also applied. Briefly, the FFQ included 133 food items in the following categories: dairy products, carbohydrates, fats, proteins, vegetables, fruits, water, beverages with and without added sugar and highly processed calorie-dense foods (i.e., sweetened beverages, candies, fast food, cakes, etc., were adapted for the pediatric population) [25]. This FFQ has been previously validated and used to assess and inform dietary habits in the Mexican population [26]. For this study, the reported raw data were used to estimate the total dairy intake (TDI) and DPI.

##### Dairy Serving Intake

Total daily energy intake (TDEI) was calculated using the Food Processor Software version 11.1^®^ (ESHA research, Oak Brook, IL, USA), and, for local products, we used the published Mexican equivalents [27]. The DPI was collected by the FFQ. The specific categories of dairy products included were whole milk, low-fat and skim milk, Manchego cheese, Oaxaca cheese, fresh cheese, ice cream, yogurt with sugar, flavored cheese (industrialized sweetened cheese), sweet milk (whole milk with added sugar) and flavored milk (industrialized sweetened milk), and were analyzed as number of serving intakes as well as ml/day or g/day. Detailed nutritional contents of these products can be found in Supplementary Table S1.

Dairy intake was categorized according the DGA 2020–2025, where adequate intake is 2 ½ cups per day (600 mL or 20 oz) for children aged 2 to 8 years and 3 cups per day (720 mL or 24 oz) for children and adolescents aged 9 to 18 years [6].

Misreporting bias was identified via applying the Goldberg cut-off method adapted for children by Black [28] and previously published by our group. Briefly, 15% of the sample was classified as over-reporting, 3% as under-reporting and 81% as plausible reports [25].

Dairy products without added sugar, sodium or excess saturated fat were classified as good quality (DGQ); and those with added sugar, excess sodium (sodium ≥ 1 mg/kcal or ≥300 mg) or excess saturated fat (≥10% of total energy) as poor quality (DPQ) [29].

#### 2.3.3. Physical Activity and Lifestyle Factors

We applied two questionnaires to assess the frequency, duration and intensity of physical activity and other lifestyle factors. The first was applied as a structured interview with direct questions based on the National Survey of Health and Nutrition (ENSANUT). It included questions about specific physical activities, such as structured exercise classes (e.g., swimming, soccer, basketball), as well as recreational physical activities, like playing in the park, riding a bicycle, dancing and daily life activities such as walking, doing laundry or sweeping. Lifestyle factor questions included questions on sleep hours on weekdays and weekends (i.e., the time the participants go to sleep and the time they awaken on a school day and on a weekend day) and daily time dedicated to watching television or playing with a computer, tablet or cell phone or playing videogames (on a school day and on a weekend day) [30,31].

The second questionnaire was the Spanish version of the self-administered International Physical Activity Questionnaire (IPAQ) and was administered under the parent’s or legal guardian’s supervision for children > 8 years and answered by parents for children ≤ 7 years.

### 2.4. Statistical Analyses

Descriptive statistics are used to report the demographics and general characteristics of the sample. Values are expressed as means, standard deviations of the mean or absolute numbers and percentages for normally distributed variables, or medians and interquartile ranges (IQR) for variables with non-parametric distribution.

A non-parametric statistical test was conducted to compare the median between groups. Given the non-normal distribution of the dairy product intake data, the Mann–Whitney U test (for two independent groups) or Kruskal–Wallis test, followed by post hoc pairwise comparisons with Bonferroni correction when appropriate, was applied to assess statistically significant differences in the median values.

Simple linear regressions were performed to test the independent effect of dairy product intake with FMI, LMI and BMC. And with adjustments for age, sex physical activity, sleep time and screen hours. Multiple linear regression analyses were performed to test the independent effect of the daily intake of each dairy product on the FMI, LMI and BMC.

All data were analyzed using SPSS IBM^®^ software (version 23.0) without replacement of missing values. Statistical significance was set to the value of *p* < 0.05.

## 3. Results

We included 1840 urban Mexican mestizo children and adolescents aged 4 to 18 years. Of the sample, 52% was female, 16% of the participants were classified as having obesity and 16% with overweight. Clinical and demographic characteristics are summarized by age group in Table 1. A flowchart of the included subjects is shown in Supplementary Figure S1.

The mean DPI for the total sample was 4.0 ± 2.4 servings/day (from beverages: 426 ± 266 mL/day, and from solids: 31.4 ± 29.2 g/day), with DGQ being the major source (2.3 ± 1.4 serving/day vs. DPQ 1.7 ± 1.6 serving/day), with no difference between age or sex groups.

Fifty-seven percent of children and adolescents (n = 1042) met the ADG 2020–2025 recommendations with a mean of 4.3 ± 2.4 servings/day (consisting of 632 mL ± 409 mL/day and 32.2 g ± 30.8 g/day and 2.5 ± 1.5 servings/day of DGQ and 1.8 ± 1.6 servings/day of DPQ). For those subjects not complying with ADG 2020–2025 recommendations, the mean TDI was 3.6 ± 2.3 servings/day (consisting of 478 mL ± 391 mL/day and 30.2 g ± 26.8 g/day and 2.0 ± 1.4 servings/day of DGQ and 1.6 ± 1.5 servings/day of DPQ).

DPI is described in detail in Table 2. Whole milk represented the most consumed dairy product in all age groups. Adolescents showed a lower average daily DPI (servings/day) compared to school-aged children, but this was not statistically significant. The patterns of DPI between sexes within each age group were generally similar. Some statistically significant differences were observed; however, they were not clinically relevant, as the overall intake of such products was marginal among participants. Similarly, when comparing DPI across BMI categories—underweight, normal weight, overweight and obese—within each age group, no significant differences were observed, as shown in Supplementary Table S2.

When analyzing the association between DPI and the BC of participants, DPI quantity was found to have a statistically significant inverse relationship with the FMI, as shown in Table 3. For each dairy serving, the FMI decreased by 0.11 kg/m^2^ (beta = −0.11 kg/m^2^ 95% CI −0.17 to −0.05, *p* < 0.001), and remained significant after adjusting for age, sex, physical activity, sleep time and screen hours (beta = −0.07 kg/m^2^ 95% CI −0.14 to −0.02; *p* = 0.005). A similar relationship was found for the LMI (beta = −0.07 kg/m^2^ −0.10 to −0.02; *p* = 0.003); however, when adjusted for age, sex, physical activity, sleep time and screen hours, statistical significance was lost (beta = −0.03, 95% CI −0.06 to 0.001; *p* = 0.061), as shown in Table 4. We did not find any association between DPI and BMC or bone mineral density (BMD) for total body less head (TBLH) or lumbar spine (L1–L4) (shown in Supplementary Tables S3–S6).

We also assessed DGQ intake and found it had a higher inverse relationship with the FMI (beta −0.17 95% CI −0.26 to −0.07; *p* < 0.001) that also remained significant after adjusting for age, sex and level of physical activity (beta −0.13 95% CI −0.22 to −0.03, *p* = 0.008). For each good-quality dairy serving, the FMI decreased by 0.17 kg/m^2^ (95% CI −0.26 to −0.07 *p* < 0.001).

In order to evaluate the association of each type of dairy product with the FMI and LMI, we performed a multiple regression analysis, and documented significant inverse relationships between whole milk intake and the FMI (beta = −0.16 kg/m^2^, 95% CI −0.29 to −0.04; *p* = 0.011), and between flavored cheese and flavored milk intake and the LMI (beta = −0.62 kg/m^2^, 95% CI −1.0 to −0.24; *p* = 0.001 and beta −0.22 kg/m^2^, 95% CI −0.39 to −0.04; *p* = 0.016, respectively), as shown in Table 5 and Table 6. When age, sex, physical, activity sleep time and screen hours were considered for the adjusted beta coefficients between whole milk intake and FMI, the association remained (beta = −0.15 kg/m^2^, 95% CI −0.27 to −0.02; *p* = 0.026). However, when age, sex, physical activity, sleep time and screen hours were considered for the LMI, the adjusted beta coefficients lost statistical significance.

## 4. Discussion

In recent years, Mexico—like many other countries—has undergone a significant nutritional transition marked by the decline of traditional, nutrient-dense dietary patterns and the increasing incorporation of ultra-processed foods, particularly among children and adolescents [32]. Dairy products (DPs), historically recognized for their role in providing high-quality protein, calcium and other essential micronutrients, have not been exempt from this shift. Our findings highlight two concerning trends: a general reduction in total DP consumption and a qualitative deterioration in the types of DPs being consumed characterized by an increased intake of flavored, sweetened beverages and ultra-processed dairy analogs.

Previous national surveys and regional studies have similarly reported reductions in milk intake across Mexican households, particularly among lower-income groups. Rivera et al. described how dairy consumption in Mexico has declined over the past two decades, while sweetened beverages and processed snacks have expanded their dietary share [33]. Our findings build upon this literature by demonstrating that not only is dairy intake declining, but the nutritional quality of the dairy products consumed is worsening. This trend is particularly evident among children and adolescents—a population highly vulnerable to long-term metabolic programming [34].

This shift is concerning given the well-established role of dairy in supporting growth and development during childhood and adolescence, when calcium, vitamin D and protein requirements are elevated. Multiple studies in low- and middle-income countries have shown that dairy intake is positively associated with linear growth, bone mineral content and lean mass and inversely associated with stunting and sarcopenic adiposity [35,36]. When dairy is displaced by ultra-processed substitutes, these benefits may not only be diminished but also replaced by increased risk factors for obesity and cardiometabolic dysfunction [37].

In our study, the observed pattern of low-quality dairy consumption was significantly associated with increased adiposity—measured by the fat mass index (FMI)—and reduced lean mass index (LMI) values among Mexican children and adolescents. These associations are biologically plausible and consistent with the existing evidence indicating that diets low in high-quality animal protein, such as that found in traditional milk and yogurt, may impair lean tissue development during critical periods of growth [36,38]. Concurrently, frequent consumption of sugar-rich, low-protein dairy-like beverages may promote excess energy intake and insulin-driven lipogenesis, contributing to body fat accumulation and dysregulated growth patterns [39].

Notably, these relationships responded differently when adjusted for lifestyle factors. The association with the FMI remained statistically significant after controlling for age, sex, sleep duration, screen time and physical activity, suggesting an independent contribution of nutritionally poor dairy products to adiposity accumulation. In contrast, the association with the LMI was attenuated and lost statistical significance in adjusted models, indicating that lean tissue development may be more sensitive to the combined effects of dietary quality, energy expenditure and other behavioral determinants [40]. These findings underscore the complex interplay between diet and lifestyle in shaping pediatric body composition and highlight the importance of considering co-exposures in nutritional risk assessment.

Notably, we did not detect any significant association between dairy intake and bone mineral content (BMC). While this may seem counterintuitive given dairy’s well-established role in supporting skeletal health, it aligns with evidence that bone accretion in children is influenced by multiple factors—including genetics, hormonal status, mechanical loading and pubertal development—in addition to dietary intake [35,41,42,43]. Furthermore, the cross-sectional design of our study and the lack of longitudinal dietary exposure or pubertal staging data may have limited our ability to detect longer-term associations between dairy intake and BMC.

A major strength of our study lies in the use of dual-energy X-ray absorptiometry (DXA) to assess body composition. Unlike anthropometric measures such as BMI or skinfold thickness, which offer only indirect or compartment-limited estimates, DXA provides validated, compartment-specific measurements of fat mass, lean mass and bone mineral content. The use of DXA enhances the internal validity of our findings and improves comparability with international data, especially in pediatric populations undergoing dynamic changes in growth and development [44,45].

Our study is not without limitations. Its cross-sectional nature precludes causal inference, and the reliance on self-reported dietary intake and lifestyle behaviors may have introduced measurement error or recall bias. However, the biological plausibility of our findings and their consistency with prior research strengthen their interpretability. Further longitudinal studies are warranted to explore the long-term effects of both the quantity and nutritional quality of dairy intake on growth and body composition outcomes in Mexican youth.

Importantly, our results highlight the need for public health policies and messaging to go beyond the promotion of dairy intake in general. Greater emphasis must be placed on the nutritional quality of dairy products—distinguishing traditional options such as plain milk, yogurt and cheese from sugar-sweetened dairy beverages and ultra-processed alternatives with limited protein and micronutrient content. This distinction is critical for school-based nutrition programs and social assistance schemes, which frequently include dairy as a staple food group. Without more rigorous standards for nutritional quality, such programs risk inadvertently reinforcing poor dietary patterns [46].

## 5. Conclusions

In conclusion, the dietary pattern identified—marked by reduced intake of milk-derived products and increased consumption of nutritionally poor substitutes—is associated with unfavorable body composition outcomes in children and adolescents. These findings call for targeted nutritional strategies and policy interventions that preserve access to high-quality dairy products while minimizing the availability and consumption of low-nutrient alternatives in populations undergoing critical stages of development.

## Figures and Tables

**Table 1:** Demographic and clinical data by age group.

	Children	Children	Adolescents	Total Sample
	4–8 Years	9–13 Years	14–18 Years	4–18 Years
	(n = 610, 33.2%)	(n = 703, 38.2%)	(n = 527, 28.6%)	(n = 1840)
Age (years) mean ± S.D.	7.0 ± 1.2	11.4 ± 1.5	15.9 ± 1.1	11.4 ± 1.5
Male n (%)	330 (54%)	374 (53%)	253 (48%)	957 (52%)
Female n (%)	280 (46%)	329 (47%)	274 (52%)	883 (48%)
Weight (kg) mean ± S.D.	24.7 ± 7.2	43.6 ± 13.5	59.5 ± 12.3	41.9 ± 17.8
Height (cm) mean ± S.D.	120.1 ± 9.3	146.0 ± 10.9	162.8 ± 8.2	142.2 ± 19.6
Height z-score mean ± S.D.	−0.27 ± 1.03	−0.15 ± 1.02	−0.59 ± 0.92	−0.31 ± 1.01
BMI (kg/m^2^) mean ± S.D.	16.9 ± 3.0	20.1 ± 4.3	22.4 ± 4.1	19.7 ± 4.4
BMI z-score mean ± S.D.	0.27 ± 1.26	0.44 ± 1.19	0.32 ± 1.05	0.35 ± 1.18
Waist circumference (cm) mean ± S.D.	57.6 ± 8.3	69.6 ± 11.4	76.0 ± 9.4	67.5 ± 12.3
Sexual maturity rating Tanner stage				
1	595 (98%)	239 (34%)	0	834 (45%)
2	15 (2%)	204 (29%)	4 (1%)	222 (12%)
3	0	176 (25%)	61 (12%)	239 (13%)
4	0	81 (12%)	278 (53%)	358 (20%)
5	0	3 (0.4%)	184 (35%)	187 (10%)
BMI category				
Healthy weight n (%)	404 (66%)	418 (59%)	362 (69%)	1184 (64%)
Overweight n (%)	78 (13%)	115 (16%)	93 (18%)	286 (16%)
Obesity n (%)	91 (15%)	139 (20%)	56 (11%)	286 (16%)
Low weight n (%)	37 (6%)	31 (4%)	16 (3%)	84 (5%)
Body composition variables				
DXA BMC (kg) mean ± S.D.	0.86 ± 0.18	1.51 ± 0.38	2.26 ± 0.37	1.51 ± 0.64
DXA FM (kg) mean ± S.D.	7.7 ± 4.0	14.8 ± 7.5	18.3 ± 7.9	13.4 ± 8.0
DXA LM (kg) mean ± S.D.	16.1 ± 3.3	27.1 ± 7.0	38.9 ± 7.6	26.9 ± 10.9
DXA FM% mean ± S.D.	29.7 ± 7.0	32.4 ± 8.3	30.0 ± 9.4	30.8 ± 8.4
Fat mass index (kg/m^2^) mean ± S.D.	5.2 ± 2.2	6.8 ± 3.0	7.0 ± 3.1	6.3 ± 2.9
Lean mass index (kg/m^2^) mean ± S.D.	11.0 ± 1.0	12.5 ± 1.7	14.6 ± 1.9	12.6 ± 2.1
Lean/fat mass index (kg/m^2^) mean ± S.D.	2.4 ± 0.8	2.2 ± 0.9	2.6 ± 1.4	2.4 ± 1.0
Dairy product intake				
DPI (serving/day) mean ± S.D.	4.3 ± 2.4	4.0 ± 2.4	3.7 ± 2.2	4.0 ± 2.4
DPI liquid (mL/day) mean ± S.D.	465 ± 244	418 ± 264	393 ± 288	426 ± 266
DPI solid (g/day) mean ± S.D.	31.8 ± 28.1	31.9 ± 31.5	30.1 ± 27.0	31.4 ± 29.2
DGQ DPI (serving/day) mean ± S.D.	2.3 ± 1.4	2.3 ± 1.5	2.3 ± 1.4	2.3 ± 1.4
DPQ DPI (serving/day) mean ± S.D.	1.6 ± 1.6	1.4 ± 1.4	1.1 ± 1.2	1.7 ± 1.6
Compliant with recommendations n (%)	397 (65%)	394 (56%)	251 (48%)	1042 (57%)
Total energy intake (kcal/day)	2168 ± 1092	2555 ± 12,085	2988 ± 4064	2555 ± 2405

BMI = body mass index, FM = fat mass, LM = lean mass, BMC = bone mineral content, DPI: dairy product intake, DGQ: dairy of good quality, DPQ: dairy of poor quality. The age groups were defined according to the DGA recommendations for daily dairy intake.

**Table 2:** Mean intake of dairy products among age group categories and sex.

Dairy Product	4–8 Years			9–13 Years			14–18 Years			Total Sample
	Male (n= 330, 54%)	Female (n = 280, 46%)	*p* Value	Male (n = 374, 53%)	Female (n = 329, 47%)	*p* Value	Male (n = 253, 48%)	Female (n = 274, 52%)	*p* Value	
Whole milk (mL)	240 (16–600)	240 (102.9–600)	0.502	240 (34–600)	240 (16–600)	0.260	240 (34–600)	240 (16–600)	0.203	240 (34.3–600)
Low-fat and skim milk (mL)	0 (0–4.3)	0 (0–8.0)	0.024	0 (0–0.0)	0 (0–8.0)	0.011	0 (0–0)	0 (0–8)	0.526	0 (0–16)
Manchego Cheese (g)	2.0 (0–4.3)	2.0 (0–4.3)	0.775	2.0 (1.0–4.3)	2.0 (0–12.9)	0.959	2.0 (1.0–12.9)	2.0 (0–4.3)	0.809	2.0 (1–4.3)
Oaxaca Cheese (g)	4.3 (2.0–12.9)	4.3 (2.0–12.9)	0.202	4.3 (2.0–12.9)	4.3 (2.0–12.9)	0.119	4.3 (2.0–12.9)	4.3 (2.0–12.9)	0.328	4.3 (2.0–12.9)
Fresh cheese (g)	4.3 (1.0–12.9)	4.3 (1.0–12.9)	0.799	4.3 (1.0–12.9)	4.3 (1.0–12.9)	0.714	4.3 (1.0–12.9)	4.3 (1.0–12.9)	0.094	4.3 (1.0–12.9)
Ice cream (g)	6.0 (3.0–12.9)	6.0 (3.0–6.0)	0.959	6.0 (3.0–6.0)	6.0 (3.0–12.9)	0.213	6.0 (3.0–6.0)	6.0 (3.0–12.9)	0.487	6 (3.0–6.0)
Yogurt with sugar (mL)	17.9 (8.3–53.6)	17.9 (8.3–53.6)	0.478	17.9 (8.3–53.6)	17.9 (8.3–53.6)	0.957	17.9 (8.3–53.6)	17.9 (8.3–53.6)	0.624	17.9 (8–103)
Sweetened probiotic milk beverage (mL)	11.4 (2.7–34.3)	11.4 (2.7–34.3)	0.725	11.4 (2.7–34.3)	5.3 (2.7–34.3)	0.237	5.3 (2.7–11.4)	5.3 (2.7–34.3)	0.016	5.3 (2.3–34.3)
Flavored cheese (g)	6.7 (0–14.3)	6.7 (3.3–42.9)	0.084	3.3 (0–14.3)	3.3 (0–14.3)	0.499	3.3 (0–6.7)	3.3 (0–6.7)	0.457	3.3 (0–14.3)
Sweet milk (m)	16 (8–103)	16 (8–103)	0.990	16 (8–103)	16 (8–103)	0.963	16 (0–103)	8 (0–34)	0.064	16 (8–102.9)
Flavored milk (mL)	16 (0–103)	16 (0–103)	0.500	16 (0–103)	16 (0–103)	0.948	8 (0–34)	8 (8–16)	0.473	16 (0–34.3)
Total DPI (servings/day)	3.9 (2.6–5.6)	4.0 (2.6–5.6)	0.935	3.8 (2.2–5.2)	3.6 (2.3–4.8)	0.185	3.8 (2.3–5.1)	3.3 (1.8–4.7)	0.027	3.7 (2.3–5.2)

DPI: Dairy-product intake. Values are presented as median and interquartile range (IQR). Differences between groups were assessed using the Mann–Whitney U test.

**Table 3:** Linear regression analysis of DPI (servings/day) with FMI (kg/m^2^), simple and adjusted for age, sex, physical activity, sleep time and screen hours.

Simple Model	Beta	95% CI	*p* Value
DPI (servings/day)	−0.1	(−0.17 to −0.06)	<0.001
Adjusted model			
DPI (servings/day)	−0.07	(−0.14 to −0.02)	0.005
Age (years)	0.18	(0.10 to 0.19)	<0.001
Sex (female)	0.23	(1.07 to 1.62)	<0.001
Physical activity (h)	−0.13	(−0.17 to −0.08)	<0.001
Sleep time (h)	−0.08	(−0.29 to −0.06)	0.003
Screen time (h)	0.08	(0.04 to 0.22)	0.003

DPI: Dairy Product Intake; FMI: fat mass index (kg/m^2^).

**Table 4:** Linear regression analysis of DPT (servings/day) with LMI (kg/m^2^), simple and adjusted for age, sex, physical activity, sleep time and screen hours.

Simple Model	Beta	95% CI	*p* Value
DPI (servings/day)	−0.07	(−0.10 to −0.02)	0.003
Adjusted model			
DPI (servings/day)	−0.03	(−0.06 to 0.00)	0.061
Age (years)	0.64	(0.35 to 0.40)	<0.001
Sex (female)	−0.22	(−1.09 to −0.80)	<0.001
Physical activity (h)	0.05	(0.01 to 0.06)	0.01
Sleep time (h)	−0.07	(−0.17 to −0.05)	<0.001
Screen time (h)	0.03	(−0.01 to 0.09)	0.086

DPI: Dairy Product Intake; LMI: lean mass index (kg/m^2^).

**Table 5:** Multiple linear regression between type of DPI (serving/day) and FMI.

	B	95% CI		*p* Value
Whole milk	−0.16	−0.29	−0.04	0.011
Low-fat and skim milk	−0.17	−0.33	0	0.054
Manchego Cheese	0.06	−0.28	0.41	0.715
Oaxaca Cheese	−0.13	−0.49	0.24	0.504
Fresh cheese	−0.11	−0.49	0.26	0.547
Ice cream	0.31	−0.46	1.09	0.43
Yogurt with sugar	0.03	−0.3	0.36	0.849
Sweetened probiotic milk beverage	−0.03	−0.35	0.3	0.879
Flavored cheese	−0.35	−0.88	0.17	0.183
Sweet milk	−0.18	−0.41	0.06	0.145
Flavored milk	−0.04	−0.29	0.2	0.744

FMI: Fat mass index (kg/m^2^).

**Table 6:** Multiple linear regression between type of DPI (serving/day) and LMI.

	B	95% CI		*p* Value
Whole milk	−0.03	−0.12	0.06	0.558
Low-fat and skim milk	0.1	−0.02	0.23	0.09
Manchego Cheese	0.11	−0.14	0.36	0.386
Oaxaca Cheese	−0.06	−0.32	0.21	0.675
Fresh cheese	0.02	−0.25	0.29	0.902
Ice cream	−0.01	−0.57	0.55	0.98
Yogurt with sugar	0.04	−0.19	0.28	0.721
Sweetened probiotic milk beverage	−0.07	−0.3	0.17	0.585
Flavored cheese	−0.62	−1	−0.24	0.001
Sweet milk	−0.05	−0.22	0.12	0.581
Flavored milk	−0.22	−0.39	−0.04	0.016

LMI: Lean mass index (kg/m^2^).

## Data Availability

The data that support the results of this study are not publicly available because they contain information that could compromise the privacy of the pediatric participants who participated in the research. For more information, you can contact the corresponding author.

## Supplementary Materials

The following supporting information can be downloaded at https://www.mdpi.com/article/10.3390/nu17162705/s1: Figure S1: Participant flowchart. Table S1: Nutrient composition per standard serving of dairy product. Table S2: DPI comparison between BMI categories within each age group. Table S3: Linear regression analysis of dairy product intake (portions/day) with bone mineral content (kg), simple and adjusted for age, sex, physical activity, sleep time and screen hours; Table S4: Multiple linear regression between type of dairy product intake (portion/day) and BMC. Table S5: Linear regression analysis of dairy product intake (portions/day) with lumbar bone mineral density (g/cm^2^), simple and adjusted for age, sex, physical activity, sleep time and screen hours. Table S6: Linear regression analysis of dairy product intake (portions/day) with total body bone mineral density (g/cm^2^), simple and adjusted for age, sex, physical activity, sleep time and screen hours. Table S7. DPI comparison between socioeconomical level categories within each age group.

## Abbreviations

The following abbreviations are used in this manuscript:

DPI	Dairy Product Intake
BC	Body Composition
DXA	Dual X-ray Densitometry
FMI	Fat Mass Index
LMI	Lean Mass Index
BMC	Bone Mineral Content
DGQ	Dairy Good Quality
DPQ	Dairy Poor Quality
