# Supplementary material for: Quantity and Quality of Dairy Product Intake and Their Relationship with Body Composition in Children and Adolescents from Mexico City
Source: Nutrients. 2025 Aug 21;17(16):2705. doi: 10.3390/nu17162705 (PMC12388920; doi:10.3390/nu17162705)
Supplement: Supplementary file 1 [file nutrients-17-02705-s001.zip › nutrients-3750913-supplementary.pdf]

Quantity and quality of dairy products intake and their relationship with body composition in children and adolescents from Mexico City.

Supplementary files:

Figure S1. Participants flowchart.

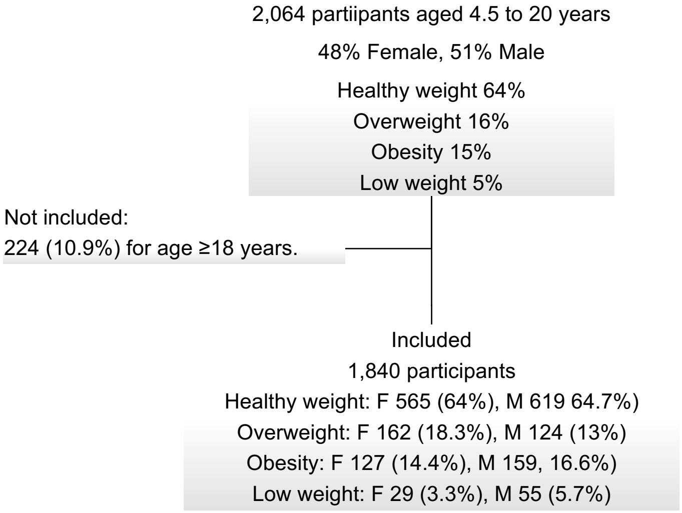

Table S1.

Table 1. Nutrient Composition per Standard Serving of Dairy Product

Dairy Product	Serving size	kcal	CHO´s	Fats	Protein	Sat Fats	Na	Added Suggar
Whole milk	240 ml	147	11	8	7	5	0.007	0
Low-fat & skim milk	240 ml	83	12	0	8	0	0.103	0
Manchego Cheese	30 g	120	0-1	10	7	6	0.300	0
Oaxaca Cheese	30 g	94	1	7	8	0	0.210	0
Fresh cheese (i.e. Panela cheese)	30 g	103	1	7	9	0	0.224	0
Ice cream	90 g	160 - 200	20 - 25	8 to 11	2 to 3	5 to 7	0.06 to 0.08	12 to 24
Yogurth with sugar	125 g	120 - 160	20 to 25	2 to 5	4 to 6	1 to 4	0.175	12 to 18
Sweetened probiotic milk beverage (i.e. Yakult®)	80 ml	55	12.7	0	1	0	0.016	10.2
Flavored cheese (i.e. Danonino®)	100 g	56-101	10 to 14	1.3-3.1	0.9-5.5	0.9	0.0381	8.6
Sweet milk	240 ml	170 to 190	24 to 28	4 to 8	7 to 8	4.5 to 5	0.150	9 to 12
Flavored milk	240 ml	180 to 220	26 to 32	4 to 8	7 to 8	4.5 to 5	0.150	15 to 22

Pérez Lizaur, A. B., Palacios González, B., Castro Becerra, A. L., & Flores Galicia, I. (2014). Sistema Mexicano de Alimentos Equivalentes (5a ed.). Fomento de Nutrición y Salud, A.C.

Table S2. DPI comparison between BMI categories within each age group.

Table S2. Mean intake of dairy products among body mass index classification by age group.

Dairy product	4 – 8 years (n= 610, 33.2%)					9 – 13 years					14 – 18 years					Total sample
	Underweight (n= 37, 6.1%)	Normal weight (n= 404, 66.2%)	Overweight (n= 78, 12.8%)	Obesity (n= 91, 14.9%)	P value	Underweight (n= 31, 4.4%)	Normal weight (n= 418, 59.5%)	Overweight (n= 115, 16.4%)	Obesity (n= 139, 19.8%)	P value	Underweight (n= 16, 3.0%)	Normal weight (n= 362, 68.7%)	Overweight (n= 93, 17.6%)	Obesity (n= 56, 10.6%)	P value	
Whole milk (mL)	189 (8 – 600)	240 (34 – 600)	240 (103 – 600)	240 (103 – 600)	0.292	240 (16 – 600)	240 (34 – 600)	240 (34 – 600)	240 (16 – 600)	0.774	420 (103 – 600)	240 (34 – 600)	240 (16 – 600)	240 (16 – 600)	0.524	240 (34.3 – 600)
Low-fat & skim milk (ml)	0 (0 – 0)	0 (0 – 8.0)	0 (0 – 0)	0 (0 – 8.0)	0.177	0 (0 – 8.0)	0 (0 – 0.0)	0 (0 – 8.0)	0 (0 – 0.0)	0.596	0 (0 – 4)	0 (0 – 0)	0 (0 – 0.0)	0 (0 – 68.6)	0.093	0 (0 – 16)
Manchego Cheese (g)	2.0 (1 – 12.9)	2.0 (0 – 4.3)	2.0 (0 – 12.9)	2.0 (0 – 4.3)	0.684	2.0 (0 – 12.9)	2.0 (1.0 – 4.3)	2.0 (0 – 12.9)	2.0 (1 – 4.3)	0.580	2.0 (1.0 – 3.1)	2.0 (1 – 12.9)	2.0 (1 – 12.9)	2.0 (0 – 4.3)	0.359	2.0 (1 – 4.3)
Oaxaca Cheese (g)	4.3 (2.0 – 12.9)	4.3 (2.0 – 12.9)	4.3 (2.0 – 12.9)	4.3 (2.0 – 12.9)	0.633	4.3 (2.0 – 12.9)	4.3 (2.0 – 12.9)	4.3 (2.0 – 12.9)	4.3 (2.0 – 12.9)	0.561	4.3 (2.0 – 12.9)	4.3 (2.0 – 12.9)	4.3 (2.0 – 12.9)	4.3 (2.0 – 12.9)	0.168	4.3 (2.0 – 12.9)
Fresh cheese (g)	4.3 (1.0 – 12.9)	4.3 (1.0 – 12.9)	4.3 (2.0 – 12.9)	4.3 (1.0 – 12.9)	0.198	12.9 (1.0 – 12.9)	4.3 (1.0 – 12.9)	4.3 (1.0 – 12.9)	4.3 (1.0 – 12.9)	0.099	2.0 (1.0 – 18.2)	4.3 (1.0 – 12.9)	4.3 (1.0 – 12.9)	4.3 (1.0 – 12.9)	0.692	4.3 (1.0 – 12.9)
Ice cream (g)	6.0 (3.0 – 6.0)	6.0 (3.0 – 6.0)	6.0 (3.0 – 12.9)	6.0 (3.0 – 12.9)	0.575	6.0 (3.0 – 12.9)	6.0 (3.0 – 12.9)	6.0 (3.0 – 6.0)	6.0 (3.0 – 12.9)	0.228	6.0 (0 – 6.0)	6.0 (3.0 – 6.0)	3.0 (3.0 – 6.0)	3.0 (3.0 – 6.0)	0.828	6 (3.0 – 6.0)
Yogurth with sugar (ml)	53.6 (17.9 – 53.6)	17.9 (8.3 – 53.6)	53.6 (8.3 – 53.6)	17.9 (8.3 – 53.6)	0.091	17.9.4 (4.2 – 53.6)	17.9 (8.3 – 53.6)	17.9 (8.3 – 53.6)	17.9 (8.3 – 53.6)	0.875	13.1 (8.3 – 35.7)	17.9 (4.2 – 53.6)	17.9 (4.2 – 53.6)	17.9 (4.2 – 53.6)	0.396	17.9 (8 – 103)
Sweetened probiotic milk beverage (ml)	5.3 (2.7 – 34.3)	11.4 (2.7 – 34.3)	11.4 (2.7 – 34.3)	11.4 (2.7 – 34.3)	0.550	5.3 (2.7 – 34.3)	8.4 (2.7 – 34.3)	5.3 (2.7 – 34.3)	11.4 (2.7 – 34.3)	0.853	5.3 (2.7 – 5.3)	5.3 (2.7 – 11.4)	5.3 (2.7 – 34.3)	5.3 (1.3 – 34.3)	0.329	5.3 (2.3 – 34.3)
Flavored cheese (g)	6.7 (0 – 42.9)	6.7 (3.3 – 14.3)	6.7 (0 – 42.9)	6.7 (3.3 – 14.3)	0.812	3.3 (0 – 14.3)	3.3 (0 – 14.3)	3.3 (0 – 14.3)	6.7 (0 – 14.3)	0.735	3.3 (0 – 5)	3.3 (0 – 6.7)	3.3 (0 – 6.7)	3.3 (0 – 14.3)	0.162	3.3 (0 – 14.3)
Sweet milk (m)	8 (0 – 34.3)	16 (0 – 103)	34.3 (8 – 188.6)	34.3 (8 – 103)	0.126	34.3 (0 – 240)	16 (0 – 103)	16 (8 – 103)	16 (0 – 103)	0.273	16 (0 – 171.4)	16 (0 – 103)	8 (0 – 34.3)	16 (4 – 68.6)	0.89	16 (8 – 102.9)
Flavored milk (ml)	8 (0 – 34.3)	16 (0 – 103)	16 (8 – 103)	16 (8 – 103)	0.898	16 (0 – 188.6)	16 (0 – 34)	8 (0 – 34)	16 (8 – 34)	0.241	8 (0 – 68.6)	8 (0 – 34)	8 (0 – 34)	8 (0 – 103)	0.677	16 (0 – 34.3)
Total dairy products intake (servings/day)	3.6 (1.8 – 5.0)	4.0 (2.7 – 5.7)	4.1 (3.0 – 5.7)	3.3 (2.2 – 4.9)	0.139	4.4 (1.4 – 7.7)	3.7 (2.4 – 5.1)	3.4 (2.2 – 4.8)	3.6 (1.8 – 4.8)	0.486	3.3 (2.0 – 4.7)	3.5 (2.0 – 4.7)	3.6 (2.4 – 5.1)	3.5 (2.0 – 5.0)	0.946	3.7 (2.3 – 5.2)

Values are presented as median and interquartile range(IQR). Median comparison across groups was conducted using the Kruskal–Wallis test.

Table S3. Linear regression analysis of DPI (servings/day) with Bone Mineral Content (kg) simple and adjusted for age, sex, physical activity, sleep time and screen hours.

**Simple model**	**Beta**	**95% CI**	**P value**
Dairy products intake (servings/day)	-0.02	(-0.04 to -0.01)	< 0.001
Adjusted model			
Dairy consumption (servings/day)	-0.02	(-0.01 to 0.00)	.156
Age (y)	0.87	(0.15 to 0.16)	< 0.001
Sex (female)	-0.08	(-0.13 to -0.07)	< 0.001
Physical activity (hr)	0.02	(0.00 to 0.01)	0.029
Sleep time (hr)	-0.04	(-0.03 to -0.01)	0.001
Screen time (hr)	0.02	(0.00 to 0.02)	0.029

DPI: Dairy Product Intake.

Table S4. Multiple linear regression between type of dairy product intake (portion/day) with BMC.

Type of dairy product	B	CI 95%		P value
Whole milk	-0.003	-0.03	0.02	0.835
Low-fat & skim milk	0.02	-0.02	0.05	0.339
Manchego Cheese	0.01	-0.06	0.09	0.745
Oaxaca Cheese	-0.03	-0.11	0.05	0.531
Fresh cheese	0.05	-0.03	0.13	0.230
Ice cream	-0.05	-0.22	0.12	0.535
Yogurth with sugar	0.03	-0.04	0.10	0.444
Yakult_FFQ	-0.05	-0.12	0.02	0.174
Flavored cheese	-0.27	-0.38	-0.15	0.000
Sweet milk	-0.03	-0.08	0.02	0.277
Flavored milk	-0.07	-0.13	-0.02	0.008

Bone mineral content (kg)

Table S5. Linear regression analysis of DPI (servings/day) with L1-L4 bone mineral density (g/cm2) simple and adjusted for age, sex, physical activity, sleep time and screen hours.

**Simple model**	**Beta**	**95% CI**	**P value**
Dairy products intake (servings/day)	-0.11	(-0.17 to -0.06)	< 0.001
Adjusted model			
Dairy consumption (servings/day)	-0.03	(0.00 to 0.00)	.051
Age (y)	0.80	(0.05 to 0.05)	< 0.001
Sex (female)	0.15	(0.05 to 0.08)	< 0.001
Physical activity (hr)	0.04	(0.00 to 0.01)	0.002
Sleep time (hr)	-0.03	(-0.01 to 0.00)	0.030
Screen time (hr)	0.04	(0.00 to 0.01)	0.008

DPI: Dairy Product Intake.

Table S6. Linear regression analysis of DPI (servings/day) with Total body bone mineral density (g/cm2) simple and adjusted for age, sex, physical activity, sleep time and screen hours.

**Simple model**	**Beta**	**95% CI**	**P value**
Dairy products intake (servings/day)	-0.11	(-0.17 to -0.06)	< 0.001
Adjusted model			
Dairy consumption (servings/day)	-0.02	(0.00 to 0.00)	.116
Age (y)	0.86	(0.04 to 0.04)	< 0.001
Sex (female)	0.01	(0.00 to 0.01)	0.405
Physical activity (hr)	0.04	(0.00 to 0.00)	< 0.001
Sleep time (hr)	-0.04	(-0.01 to 0.00)	0.002
Screen time (hr)	0.02	(0.00 to 0.01)	0.065

DPI: Dairy Product Intake.

Table S7. DPI comparison between Socioeconomical level categories within each age group.

Supplementary Table S7. Mean intake of dairy products among Socioeconomic level by age group categories.

Dairy product	4 – 8 years (292, 39.7%)				9 – 13 years (234, 31.8%)				14 – 18 years (209, 28.4%)			
	Low (n= 120, 41.1%)	Middle (n= 157, 53.8%)	Upper (n= 15, 5.1%)	P value	Low (n= 101, 43.2%)	Middle (n= 120, 17.1%)	Upper (n= 13, 1.8%)	P value	Low (n= 89, 42.6%)	Middle (n= 115, 55%%)	Upper (n= 5, 2.4%)	P value
Whole milk (mL)	240 (16 – 600)	240 (16 – 600)	600 (240 – 600)	0.341	189 (16 – 600)	240 (16 – 600)	240 (8 – 600)	0.228	240 (103 – 600)	240 (16 – 600)	600 (189 – 600)	0.131
Low-fat & skim milk (ml)	0 (0 – 0.0)	0 (0 – 8.0)*	0 (0 – 0.0)	**0.029**	0 (0 – 8.0)	0 (0 – 0.0)	0 (0 – 16.0)	0.785	0 (0 – 8)	0 (0 – 8)	0 (0 – 0)	0.227
Manchego Cheese (g)	2.0 (0 – 4.3)	2.0 (0 – 12.9)	2.0 (1 – 4.3)	0.603	2.0 (0 – 4.3)	2.0 (0 – 4.3)	12.9 (2 – 12.9)	0.110	2.0 (0 – 4.3)	2.0 (0 – 12.9)	4.3 (0 – 12.9)*	**0.027**
Oaxaca Cheese (g)	4.3 (2.0 – 12.9)	4.3 (4.3 – 12.9)	4.3 (4.3 – 12.9)	0.446	4.3 (2.0 – 12.9)	4.3 (2.0 – 12.9)	12.9 (4.3 – 23.6)	0.218	4.3 (2.0 – 12.9)	4.3 (2.0 – 12.9)	12.9 (4.3 – 12.9)	0.370
Fresh cheese (g)	4.3 (1.5 – 12.9)	4.3 (2.0 – 12.9)	4.3 (2.0 – 12.9)	0.332	4.3 (1.0 – 12.9)	4.3 (1.0 – 12.9)	4.3 (2.0 – 12.9)	0.636	4.3 (1.0 – 12.9)	4.3 (2.0 – 12.9)	12.9 (4.3 – 12.9)	0.113
Ice cream (g)	6.0 (3.0 – 6.0)	6.0 (3.0 – 12.9)	3.0 (3.0 – 6.0)	0.343	3.0 (3.0 – 6.0)	6.0 (3.0 – 6.0)	3.0 (3.0 – 6.0)	**0.001**	6.0 (3.0 – 6.0)	3.0 (3.0 – 6.0)	3.0 (3.0 – 3.0)	0.956
Yogurth with sugar (ml)	17.9 (8.3 – 53.6)	17.9 (8.3 – 53.6)	17.9 (8.3 – 53.6)	0.728	17.9 (8.3 – 53.6)	17.9 (8.3 – 53.6)	17.9 (4.2 – 53.6)	0.650	17.9 (8.3 – 53.6)	17.9 (8.3 – 53.6)	53.6 (53.6 – 98.2)*	**0.017**
Sweetened probiotic milk beverage (ml)	5.3 (2.7 – 34.3)	5.3 (2.7 – 34.3)	11.4 (0 – 34.3)	0.360	5.3 (0 – 11.4)	11.4 (2.7 – 34.3)*	5.3 (0 – 34.3)	**0.043**	5.3 (2.7 – 34.3)	5.3 (2.7 – 34.3)	34.3 (34.3– 62.9)*	**0.000**
Flavored cheese (g)	3.3 (0 – 42.9)*	3.3 (0 – 14.3)	3.3 (0 – 14.3)	**0.039**	3.3 (0 – 6.7)	3.3 (0 – 14.3)	0 (0 – 6.7)	0.682	3.3 (0 – 6.7)	3.3 (0 – 14.3)	6.7 (3.3 – 14.3)*	**0.011**
Sweet milk (m)	16 (8 – 103)	16 (8 – 103)	8 (0 – 16)*	**0.004**	16 (8 – 34.3)	16 (8 – 103)	8 (0 – 16)	0.313	16 (0 – 34)	8 (0 – 34)	16 (16 – 103)	0.293
Flavored milk (ml)	16 (0 – 103)	16 (0 – 103)	8 (0 – 34)	0.258	16 (0 – 34)	16 (8 – 34)	8 (0 – 16)	0.382	16 (0 – 34)	8 (0 – 16)	34 (16 – 103)	0.156
Total dairy products intake (servings/day)	4.0 (2.9 – 5.4)	3.9 (2.3 – 5.3)	3.5 (1.7 – 4.5)	0.578	3.8 (2.2 – 5.2)	3.6 (2.3 – 4.8)	3.9 (2.9 – 4.7)	**0.002**	3.7 (2.8 – 4.5)	3.7 (2.1 – 5.1)	3.8 (3.6 – 6.0)	0.101

Values are presented as median and interquartile range(IQR). Differences between groups were assessed using the Mann–Whitney U test.
